# Phenomenological characteristics of autobiographical future thinking in nurses with burnout: a case-control study

**DOI:** 10.3389/fpsyg.2023.1216036

**Published:** 2023-10-10

**Authors:** Bowen Xue, Yaping Feng, Jie Zheng, Xin Li, Yihui Zhao, Xiaoshan Yang, Yu Zhang, Shujin Wang, Zhiguo Hu, Hong Luo

**Affiliations:** ^1^School of Nursing, Hangzhou Normal University, Hangzhou, China; ^2^Affiliated Mental Health Center & Hangzhou Seventh People’s Hospital, Zhejiang University School of Medicine, Hangzhou, China; ^3^Affiliated Hospital of Hangzhou Normal University, Hangzhou, China; ^4^School of Nursing, Shanxi Medical University, Taiyuan, China

**Keywords:** burnout, nurses, future thinking, Sentence Completion for Events in the Future Test, work-related stress

## Abstract

**Objective:**

Nurses constitute the largest group of healthcare workers worldwide, and job burnout is very common among them. This study aims to explore abnormal future thinking in nurses with burnout. Additionally, the study investigates whether these manifestations worsen as burnout progresses.

**Methods:**

The study was conducted in inpatient ward nurses at a tertiary hospital in Hangzhou, China. In the first phase, two group of nurses were recruited: nurses with burnout (*N* = 70) and nurses without burnout (*N* = 70). In the second phase, three groups were recruited according to the burnout levels: mild burnout (*N* = 43), moderate burnout (*N* = 42) and severe burnout (*N* = 43). Data on job burnout were obtained using the Chinese Maslach Burnout Inventory. The Sentence Completion for Events in the Future Test (SCEFT) was employed to measure the content of future thinking, which was evaluated by two raters in terms of the specificity, emotional valence, and concrete content of the imagined future events. The proportions of specific types of events among all the produced events were calculated.

**Results:**

The results revealed that nurses with burnout, compared to nurses without burnout, imagined fewer specific future events, positive events, and events related to relationships and achievement. They also had more omissions. As the level of burnout increased, their impairment in future thinking worsened. Furthermore, the results also revealed that the scores of emotional exhaustion, depersonalization, and personal accomplishment had significant correlations with the proportions of positive events and events related to relationships and achievement/mastery in nurses’ future thinking content.

**Conclusion:**

The future thinking ability of nurses with burnout was impaired, and this impairment worsened as the symptoms of burnout progressed. The findings of the present study have important implications for nurse caring and advocate effective interventions targeting positive future thinking to mitigate nurses’ burnout.

## Introduction

In recent years, nurses have played a crucial role in the ongoing COVID-19 pandemic. Faced with the constant threat of COVID-19 infection, nurses endure significant psychological stress, making them vulnerable to burnout and other adverse outcomes (Galanis et al., 2021; Çağış and Yıldırım, 2023; Rizzo et al., 2023). A meta-analysis has revealed that one in ten nurses worldwide has reported experiencing burnout (Woo et al., 2020). High levels of burnout could lead to decreased job satisfaction, reduced worker productivity, lower levels of care, higher absenteeism and increased turnover (Borritz et al., 2006; Kakemam et al., 2021; Stewart et al., 2023), which could further exacerbate staffing shortages in healthcare organizations (Fasih Far et al., 2022). Therefore, it is necessary to address nurse burnout to maintain a stable healthcare workforce in times of crisis (Karimi et al., 2022). Specifically, it is vital to understand the impact of burnout on nurses’ occupational mental health and well-being, as well as its effects on patient care.

Burnout is a psychological response to prolonged workplace stress, characterized by emotional exhaustion, depersonalization, and diminished personal accomplishment (Maslach and Jackson, 1981; Chen et al., 2023; Chirico et al., 2023). Cross-sectional studies have connected burnout to psychological symptoms such as hopelessness (Civilotti et al., 2022) and depression (Chen et al., 2021). This suggested that burnout might be associated with the abnormality of future thinking. Future thinking refers to the capacity to envision or simulate experiences that might occur in one’s future (Schacter et al., 2008, 2017). Simulating future scenarios provides crucial functional benefits such as visionary decision-making (Sze et al., 2017), emotional regulation (Hallford et al., 2022), and the formation of intentions and plans (Schacter et al., 2017). Thus, future thinking is critical for the human well-being (Schacter and Addis, 2007; Ward, 2016).

Hopelessness, a key element of burnout, often involves anticipating a bleak future (Marchetti, 2019). As a positive psychological capital, hope is closely related to an individual’s expectations and plans for future goals, as well as the actions needed to achieve those goals (Snyder, 2002). Several studies have reported that hope and resilience is the dominant positive force in confronting the future and is linked negatively with job burnout (Pharris et al., 2022; Yıldırım et al., 2023; Yıldırım and Ashraf, 2023). In addition, burnout has a close connection with depression, and is sometimes referred to as job depression (Firth et al., 1987). Previous research has demonstrated that individuals with depression, schizophrenia, bipolar disorder, and Parkinson’s disease often exhibit abnormalities in future thinking (de Vito et al., 2012; Hallford et al., 2018). Notably, many studies have found that individuals with depression lack depth and vividness in their future thinking (Stöber, 2000; King et al., 2011). Finally, positive or negative expectations about the future significantly influence one’s behaviors. Negative expectations about the future can lead to negative reactions and contribute to the development of burnout (Koc and Bozkurt, 2017; Ahlstedt et al., 2019). All the mentioned evidences seem to indicate that job burnout might have an important influence on future thinking. Exploring the characteristics of future thinking in nurses with burnout might contribute to uncover the mechanism by which burnout occurs and develops.

Despite the importance of future thinking in understanding burnout, most studies on job burnout have focused on past and present situations. To the best of our knowledge, no study has investigated future thinking in nurses with burnout. Therefore, the present study attempts to fill this gap. We adopted the Sentence Completion for Events in the Future Test (SCEFT) to evaluate the content of future thinking, which has been used to assess future thinking in people with schizophrenia (Gan et al., 2015) and autism spectrum disorder (Crane et al., 2013). Specifically, we aim to examine whether the future thinking in nurses with burnout is impaired and to identify any specific characteristics in the content of their future thinking. Additionally, we aim to determine if these manifestations worsen as burnout progresses. We hypothesize that nurses with burnout will exhibit impaired future thinking with negatively biased imaginative content. Furthermore, we anticipate that these manifestations will intensify with the severity of burnout.

## Methods

### Study design

The study adopted a case–control design. In the first phase, two groups of nurses were recruited: those with job burnout and those without. In the second phase, nurses with burnout were categorized into three levels: mild, moderate, and severe burnout, leading to three groups. The study was conducted from March 2022 to May 2022 at a renowned tertiary hospital in eastern China.

### Participants

The sample size was calculated using G*Power version 3.1 (Franz, Universität Kiel, Germany), considering an effect size of 1 (Gan et al., 2015), an alpha error probability of 0.05, a power of 0.8, and an allocation ratio of N2/N1 of 1. As a result, the sample size for each group in the first phase should be more than 17. Initially, 500 female nurses volunteered for the study and underwent an exhaustive screening process using the Maslach Burnout Inventory (MBI) (Maslach and Jackson, 1981). In the first phase, 70 nurses who met the burnout criteria and 70 nurses who did not were recruited.

In the second phase, three groups of nurses were included, i.e., mild burnout, moderate burnout and severe burnout. The G*Power calculator showed that each group should consist of at least 5 participants. From the remaining 360 nurses, those who met the corresponding burnout criteria were selected. This resulted in 43 nurses with mild burnout, 42 with moderate burnout, and 43 with severe burnout.

Inclusion criteria of the participants were: (1) meeting the inclusion criteria for burnout/non-burnout, or mild/moderate/severe burnout; (2) having no history of other neurological disorders; (3) being able to complete the pen-and-paper questionnaire; and (4) provision of written informed consent and cooperation in the study. Exclusion criteria were: (1) nurses who were in training, during probation or internship; and (2) nurses who were not on duty during the survey period.

### Measurements

#### Maslach burnout inventory

The Chinese version of the MBI was utilized in this study, which consists of 22 items across three sub-dimensions: emotional exhaustion, depersonalization, and personal accomplishment. This scale has been validated and widely applied in China (Song et al., 2021). Each item is assessed using a 7-point Likert scale, where respondents rate from 0 (never) to 6 (every day) regarding their personal experiences (Wang et al., 2021). The Cronbach’s alpha coefficient for the Maslach Burnout Inventory in the present study was 0.737.

The criteria for identifying burnout were based on established norms among healthcare professionals (Ball et al., 2020). Specifically, participants were identified as experiencing burnout if they scored 27 or higher on emotional exhaustion, 10 or higher on depersonalization, or 33 or lower on personal accomplishment. Participants who met the criteria in at least one of the three dimensions were recruited in the burnout group in the first phase of the study. Meanwhile, participants who did not meet any of the criteria in any of the three dimensions were selected for the group without burnout. During the second phase, participants who met only one criterion out of the three dimensions were assigned to the mild burnout group. Those who met two criteria from the three dimensions were placed in the moderate burnout group. Finally, participants who met all three criteria from the three dimensions were categorized into the severe burnout group.

#### Sentence completion for events in the future test

The SCEFT includes 11 incomplete sentences. For example, “Next year, I…” “Next week, I…” “I can see clearly in the future…” Nurses were instructed to complete these sentences with their own thoughts, ensuring that each response conveyed unique content different from the others. The completed sentences were rated in three aspects. First, the items were rated according to their specificity, in which five types of events were included: specific events (with a specific time and place within a day), extended events (specific events lasting more than a day), categorical events (generally belonging to a category of events), semantic associates (semantic information), and omission (participants cannot imagine anything) (Anderson and Dewhurst, 2009). Second, the items were rated on emotional valence (i.e., positive, negative, or neutral events). Third, the items were rated according to their content, in which twelve types of events were categorized: life-threatening events, exploration/recreation, relationships, hospitalization/stigmatization, achievement/mastery, guilt/shame, drug/alcohol events, failure, happy events, career events, neutral events, and events unclassifiable (Raffard et al., 2016).

#### Data collection

Data collection for this study took place in a quiet room. Initially, participants were given an overview of the study and asked to provide informed consent. They were then requested to complete demographic information, the MBI scale, and the SCEFT task.

Research materials were collected on-site by the researchers and team members, all of whom underwent standardized training. During the evaluation of future thinking, a stringent grading protocol was followed. All raters underwent training and followed the grading procedures consistent with established research practices. Each nurse’s responses in SCEFT were independently coded and scored by two raters. In cases of disagreement on the interpretation of a certain sentence between the two raters, a third rater get involved and discussed with the previous two raters to obtain a final score. The inter-rater reliability was assessed using Cohen’s kappa, which indicated high reliability in the present study: *K* = 0.82 for the event specificity, *K* = 0.88 for the emotional valence, and *K* = 0.80 for the content.

#### Data analyses

To analyze the data, we calculated the ratio of the number of responses for each nurse’s content in terms of specificity, emotional valence, and content. For instance, if a nurse provided six positive events, one negative event, and four neutral events out of 11 sentences related to emotional valence, the scores for positive, negative, and neutral events would be calculated as 6/11 = 0.55, 1/11 = 0.09, and 4/11 = 0.36, respectively.

The statistical procedures were performed using IBM SPSS 26.0 (IBM Corp. Released, Armonk, NY, USA). Descriptive analysis, chi-square tests, and Fisher’s exact test were used to describe and compare the demographic data (education level, working seniority, employment form, professional title, and working time with patients) and indices of future thinking. Independent-samples t-tests and one-way analysis of variance (ANOVA) were used to compare the differences in nurses’ future thinking for data that conformed to a normal distribution (see Supplementary Table S1). For data that did not adhere to a normal distribution, we conducted analyses using the Kruskal-Wallis test. Tukey’s and Mann–Whitney *post hoc* test were used to assess pairwise differences in sample means. Spearman correlation analyses were conducted to explore the relationships between burnout sub-dimension and the variables concerning future thinking.

## Results

### Future thinking of nurses with and without burnout

#### Nurses’ characteristics

Both of the burnout group and no-burnout group consisted of 70 nurses. There were no significant differences in the demographic characteristics between the two groups (see Supplementary Table S2 for details).

#### Comparison of the content of imagined future events between the two groups

The proportion of events generated in each category in the SCEFT was presented in Table 1. The outcome variable was compared between the two groups (nurses with and without burnout), and the results was also showed in Table 1.

**Table 1:** Intergroup differences in the proportions of imagined future events between nurses with and without burnout.

Item	Burnout (*N* = 70)	Without burnout (*N* = 70)	*t/Z*	*P*
Specificity				
Specific events	0.18 (0.00,0.91)	0.18 (0.00,0.91)	−2.32	0.020^*^
Extended events	0.18 (0.00,0.45)	0.18 (0.00,0.91)	−0.07	0.948
Categorical events	0.00 (0.00,0.36)	0.00 (0.00,0.82)	−2.01	0.044^*^
Semantic associates	0.45 ± 0.25	0.35 ± 0.28	−0.28	0.778
Omission	0.18 (0.00,0.82)	0.00 (0.00,0.64)	−4.48	<0.001^**^
Emotional valence				
Positive	0.45 (0.00,1.00)	0.64 (0.09,1.00)	−3.10	0.002^*^
Negative	0.00 (0.00,0.45)	0.00 (0.00,0.36)	−1.73	0.084
Neutral	0.18 (0.00,0.45)	0.18 (0.00,0.55)	−1.90	0.057
Content				
Life-threatening events	0	0	/	/
Exploration/recreation	0.00 (0.00,0.18)	0.00 (0.00,0.27)	−1.28	0.201
Relationships	0.00 (0.00,0.55)	0.09 (0.00,0.55)	−6.20	<0.001^**^
Achievement/mastery	0.09 (0.00,0.55)	0.18 (0.00,0.55)	−2.73	0.006^*^
Guilt/shame	0.00 (0.00,0.09)	0.00 (0.00,0.09)	−0.57	0.572
Drug/alcohol events	0	0	/	/
Hospitalization/stigmatization	0	0	/	/
Failure	0.00 (0.00,0.09)	0.00 (0.00,0.09)	0.00	1.000
Happy events	0.20 ± 0.11	0.27 ± 0.18	3.67	<0.001^**^
Career events	0.20 ± 0.12	0.22 ± 0.12	−0.34	0.733
Neutral events	0.09 ± 0.11	0.08 ± 0.10	−3.69	<0.001^**^
Not classifiable	0.27 (0.00,0.82)	0.09 (0.00,0.36)	−4.70	<0.001^**^

For data that does not conform to a normal distribution, the form of “median (min, max)” was used to describe them. For data that conforms to a normal distribution, the “mean ± SD” was adopted to describe them. *p < 0.05. **p < 0.001.

##### Specificity

**Figure 1:**
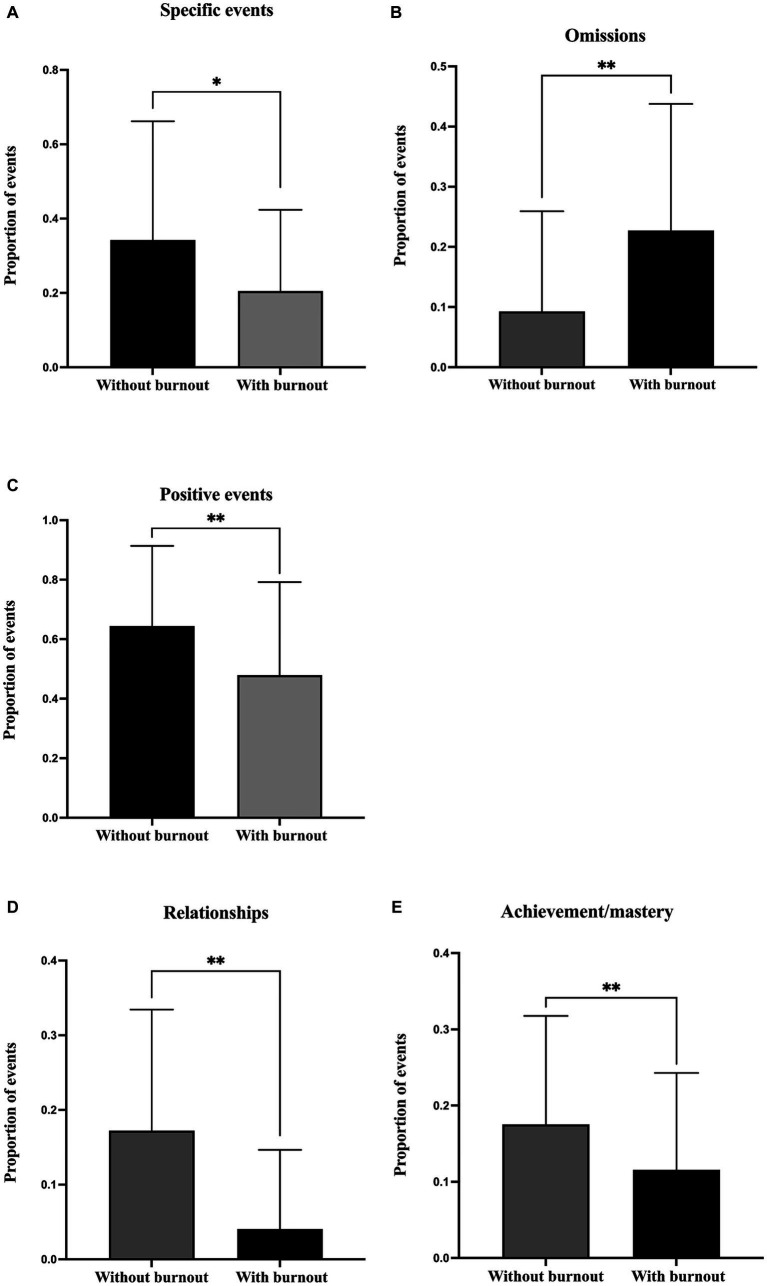
Comparison of the proportion of different categories in imagined future events between nurses with and without burnout: **(A)** specific events, **(B)** omission, **(C)** positive events, **(D)** relationships, **(E)** achievement/mastery. *p < 0.05. **p < 0.001.

In terms of specificity, imagined events were categorized into five types: specific events, extended events, categorical events, semantic associates, and omissions. The results indicated that nurses with burnout imagined significantly fewer specific events (*Z* = −2.32, *p* < 0.05) and categorical events (*Z* = −2.01, *p* < 0.05) than nurses without burnout (Figures 1A,B). Meanwhile, nurses with burnout had significantly more omissions about the future than nurses without burnout (*Z* = −4.48, *p* < 0.001). As for extended events (*Z* = 0.07, *p* > 0.05) and semantic associate events (*t* = −0.28, *p* > 0.05), there were no significant differences between the two groups.

##### Emotional valence

Regarding the emotional valence, imagined events were categorized into positive, neutral, and negative events. The results showed that, compared to nurses without burnout, nurses with burnout imagined significant less positive future events (*Z* = −3.10, *p* < 0.05), as shown in Figure 1C. However, no significant difference was found between the two groups regarding the negative (*Z* = −1.73, *p* > 0.05) and neutral events (*Z* = −1.90, *p* > 0.05).

##### Content

In terms of the concrete content of imagined future events, they were classified into 12 categories. The results revealed that, nurses with burnout imagined significantly less future events concerning relationships (*Z* = −6.20, *p* < 0.001), achievement/mastery (*Z* = −2.73, *p* < 0.05) than nurses without burnout (see Figures 1D,E). Meanwhile, nurses with burnout (compared to nurses without burnout) imagined significantly more future neutral events (*t* = −3.69, *p* < 0.001) and unclassifiable events (*Z* = −4.70, *p* < 0.001) and fewer happy events (*t* = 3.67, *p* < 0.001). However, there were no significant differences between the two groups in events related to exploration/recreation (*Z* = −1.28, *p* > 0.05), career (*t* = −0.34, *p* > 0.05), failure (*Z* = 0.00, *p* > 0.05), and guilt/shame (*Z* = −0.56, *p* > 0.05). Additionally, no participants in either group imagined future events associated with life-threatening situations, drug/alcohol use, or hospitalization/stigmatization.

### Future thinking of nurses with different levels of burnout

#### Nurses’ characteristics

To explore the effect of different burnout levels on the future thinking, we divided the nurses with burnout into three groups: 43 nurses with mild burnout, 42 nurses with moderate burnout, and 43 nurses with severe burnout. There were no significant differences in the demographic characteristics among the three groups, as shown in Supplementary Table S3.

#### Comparison of the content of imagined future events among the three groups

The proportion of events generated in each category in the SCEFT within the three groups is presented in Table 2, and the comparative results among the three groups (nurses with mild, moderate, and severe burnout) are also shown in Table 2.

**Table 2:** Differences of proportions of imagined future events among nurses with different burnout levels.

Item	Mild burnout (*N* = 43)	Moderate burnout (*N* = 42)	Severe burnout (*N* = 43)	*H/F*	*p*
Specificity					
Specific events	0.19 ± 0.25	0.15 ± 0.18	0.08 ± 0.09	3.45	0.035^*^
Extended events	0.18 (0.00,0.55)	0.18 (0.00,0.45)	0.18 (0.00,0.45)	6.87	0.032^*^
Categorical events	0.00 (0.00,0.82)	0.00 (0.00,0.45)	0.36 (0.00,0.82)	2.14	0.343
Semantic associates	0.36 (0.00,1.00)	0.36 (0.00,0.91)	0.09 (0.00,0.91)	3.27	0.195
Omission	0.04 ± 0.10	0.12 ± 0.19	0.34 ± 0.20	47.68	<0.001^**^
Emotional valence					
Positive	0.67 ± 0.30	0.62 ± 0.32	0.34 ± 0.24	16.33	<0.001^**^
Negative	0.03 ± 0.05	0.03 ± 0.07	0.06 ± 0.10	2.22	0.113
Neutral	0.18 (0.00,0.45)	0.18 (0.00,0.45)	0.36 (0.00,0.91)	16.12	<0.001^**^
Content					
Life-threatening events	0	0	/	/	/
Exploration/recreation	0.09 ± 0.09	0.08 ± 0.08	0.03 ± 0.05	8.86	<0.001^**^
Relationships	0.14 ± 0.12	0.15 ± 0.11	0.10 ± 0.10	2.59	0.079
Achievement/mastery	0.16 ± 0.12	0.16 ± 0.14	0.08 ± 0.11	6.29	0.002^*^
Guilt/shame	0	0	/	/	/
Drug/alcohol events	0	0	/	/	/
Hospitalization/stigmatization	0	0	/	/	/
Failure	0.09	0.09	0.09	0.00	1.00
Happy events	0.18 (0.00,0.64)	0.18 (0.00,0.45)	0.18 (0.00,0.36)	5.83	0.054
Career events	0.13 ± 0.13	0.16 ± 0.13	0.18 ± 0.17	1.22	0.300
Neutral events	0.05 ± 0.09	0.02 ± 0.07	0.07 ± 0.14	1.91	0.153
Not classifiable	0.18 (0.00,0.82)	0.18 (0.00,0.82)	0.18 (0.00,0.91)	3.69	0.158

For data that does not conform to a normal distribution, the form of “median (min, max)” was used to describe them. For data that conforms to a normal distribution, the “mean ± SD” was adopted to describe them. *p < 0.05. **p < 0.001.

##### Specificity

**Figure 2:**
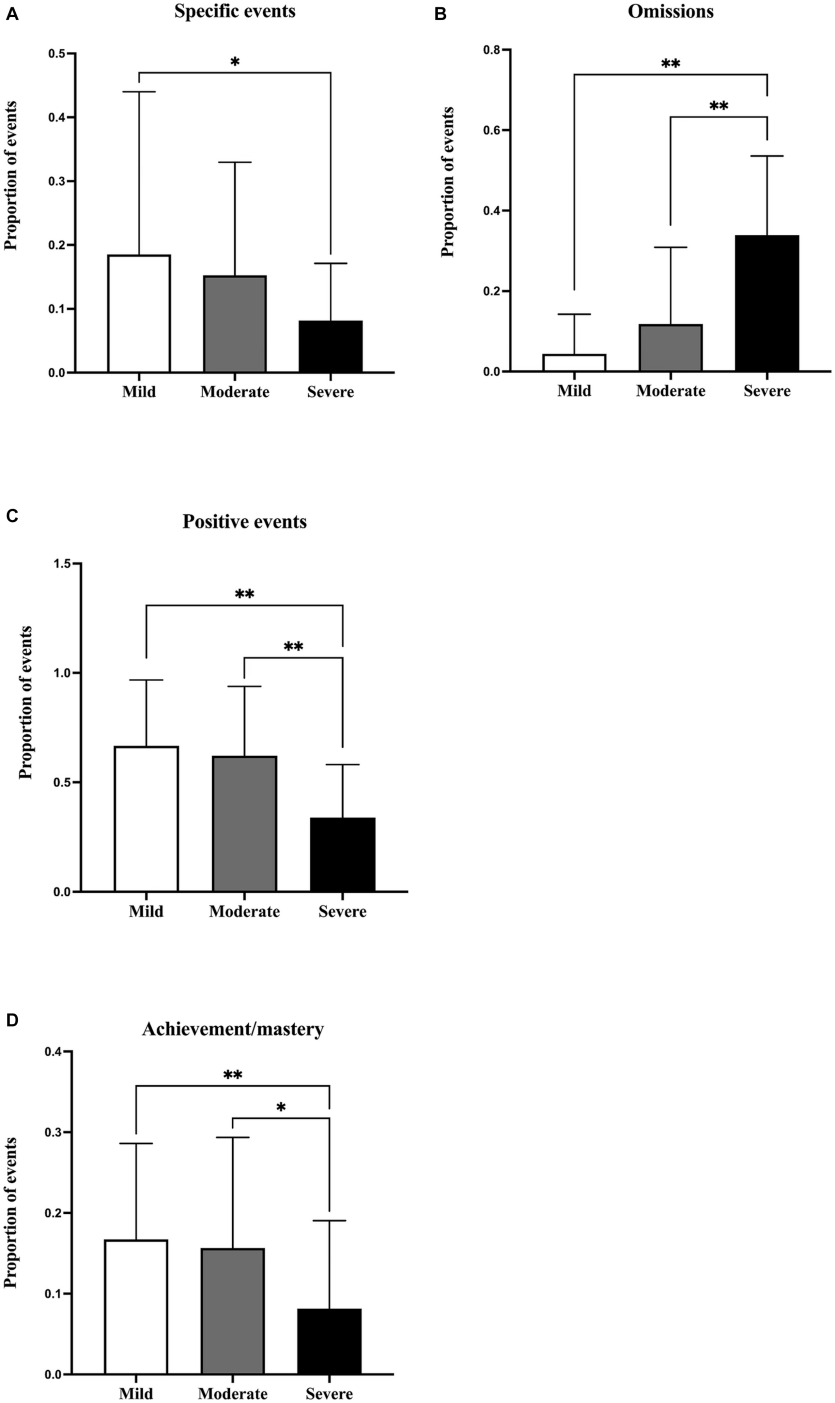
Comparison of the proportion of different categories in imagined future events among nurses with different burnout levels: **(A)** specific events, **(B)** omission, **(C)** positive events, **(D)** achievement/mastery. *p < 0.05. **p < 0.001.

Regarding the specificity, imagined future events were categorized into five types: specific events, extended events, categorical events, semantic associates, and omission. The results indicated significant differences in the proportion of specific events among the three groups [*F*(2,125) = 3.45, *p* < 0.05], as shown in Figure 2A. The Tukey’s *post hoc* test analysis showed that nurses with severe burnout imagined significant fewer specific events than nurses with mild burnout (*p* < 0.05). However, no significant difference was found between nurses with moderate burnout and other two groups (all *p*s > 0.05). For omissions, Kruskal-Wallis test showed that statistically significant differences occurred among the three groups (*H* = 47.68, *p* < 0.001). Specifically, nurses with severe burnout had significantly more omissions when imagining future events than nurses with mild and moderate burnout (all *p*s < 0.001). Meanwhile, no significant difference was found between nurses with mild and moderate burnout (*p* > 0.05), as illustrated in Figure 2B. For the extended events, the three groups also exhibited statistically significant differences (*H* = 6.87, *p* < 0.05). No significant differences were found among the three groups for the categorical events (*H* = 2.14, *p* > 0.05) and semantic associate events (*H* = 3.27, *p* > 0.05).

##### Emotional valence

In terms of the emotional valence, imagined future events were categorized as positive, neutral, or negative. The results showed significant differences in the proportion of positive events among the three groups [*F*(2,125) = 16.33, *p* < 0.001]. Nurses with severe burnout produced significant fewer positive future events than nurses with mild and moderate burnout, while no significant difference was found between nurses with mild and moderate burnout (all *p*s < 0.001), as showed in Figure 2C. For the neutral events, there was also a significant difference among the three groups (*H* = 16.121, *p* < 0.001). However, no significant difference was revealed for the negative events among the three groups [*F*(2,125) = 2.22, *p* > 0.05].

##### Content

For the specific content of the imagined future events, the three groups of nurses showed significant difference in the proportion of events concerning achievement/mastery [*F*(2,125) = 6.29, *p* < 0.05]. That is, nurses with severe burnout imagined fewer future events associated with achievement/mastery than nurses with moderate (*p* = 0.02) and mild (*p* < 0.001) burnout, while the latter two groups did not differ significantly (*p* = 0.45), as shown in Figure 2D. In addition, the results showed that there were significant difference for exploration/recreation events [*F*(2,125) = 8.86, *p* < 0.001] among the three groups. For all nurses, no events about life-threatening, guilt/shame, drug/alcohol, or hospitalization/stigmatization were reported. There were no significant differences for neutral [*F*(2,125) = 1.91, *p* > 0.05], unclassifiable (*H* = 3.69, *p >* 0.05) events, relationship [*F*(2,125) = 2.59, *p* > 0.05], failure [*F*(2,125) = 0.00, *p* > 0.05], happy events (*H* = 5.83, *p* > 0.05), and career events [*F*(2,125) = 1.22, *p* > 0.05] among the three groups.

### The relation between burnout and future thinking

To explore the relationship between the indicators of burnout and future thinking, Spearman correlations were used between the scores of the sub-dimensions (i.e., emotional exhaustion, depersonalization, and personal accomplishment) and the proportions of the certain events for which significant differences were found among groups (i.e., specific events, omissions, positive events, events on relationships, events on achievement/mastery, happy events, and neutral events). The correlation results are presented in Table 3. It was found that the scores of emotional exhaustion and depersonalization had significant negative correlations with the proportions of specific events, positive events, events on relationships, events on achievement/mastery, and happy events, while they had significant positive correlations with the proportions of neutral events and omissions. However, the score of personal accomplishment showed a reverse pattern concerning the correlations with indices of future thinking.

**Table 3:** Correlations of nurses’ burnout and future thinking.

Item	1	2	3	4	5	6	7	8	9
1	1								
2	0.81^**^	1							
3	−0.57^**^	−0.52^**^	1						
4	−0.26^**^	−0.24^**^	0.28^**^	1					
5	0.27^**^	0.32^**^	−0.42^**^	−0.39^**^	1				
6	−0.21^*^	−0.24^**^	0.34^**^	0.36^**^	−0.53^**^	1			
7	−0.29^**^	−0.38^**^	0.28^**^	0.07	−0.18^*^	0.04	1		
8	−0.17^*^	−0.19^*^	0.23^**^	−0.05	−0.26^**^	0.32^**^	−0.13	1	
9	−0.30^**^	−0.26^**^	0.34^**^	0.39^**^	−0.30^**^	0.31^**^	−0.05	0.03	1
10	0.30^**^	0.25^**^	−0.15^**^	−0.01	−0.08	−0.10	−0.15	−0.23^**^	−0.13

1 = Emotional exhaustion, 2 = Depersonalization, 3 = Personal accomplishment, 4 = Specific events, 5 = Omissions, 6 = Positive events, 7 = Relationships events, 8 = Achievement/mastery events, 9 = Happy events, 10 = Neutral event. *p < 0.05. **p < 0.001.

### Sensitivity analysis

To ensure the methodological rigor of this study, we conducted a sensitivity analysis to explore whether our main findings remained consistent under different criteria for defining burnout (Ye et al., 2008). The main results were replicated in the sensitivity analysis (See SI for the details), which indicates the credibility of our research findings.

## Discussion

Most previous studies addressing nurses with burnout have primarily explored the associated negative manifestations using scales (e.g., Barello et al., 2020; Levi et al., 2021). In the present study, we examined the features of future thinking content in nurses with burnout using a novel methodology, the SCEFT. We found that, compared to nurses without burnout, nurses with burnout had impaired imaginations of future events. Specifically, they expected less specific future events, positive events and events on relationships and achievement. With the burnout level increased, their impairment in the future thinking also worsened, as demonstrated by the results in the future thinking in nurses with mild, moderate and severe burnout. Furthermore, our study found significant correlations between the scores of emotional exhaustion, depersonalization, and personal accomplishment, and the proportions of positive events, as well as events related to relationships and achievement/mastery in nurses’ future thinking content. These results support our hypothesis that nurses’ ability of future thinking was impaired. When thinking about events in the future, nurses with burnout performed differently from nurses without burnout. They imagined a vague, less positive, or even omitted future. Similar results have been found in studies on future thinking in depressed people (Stöber, 2000; King et al., 2011). The current results provide empirical evidence that nurses with burnout experience deficits in future thinking, similar to individuals with depressive symptoms.

We found that nurses with burnout produced significantly fewer specific events than nurses without burnout. This suggested that nurses experiencing burnout demonstrated a significant impairment in constructing vivid future scenarios. This may be attributed to the influence of negative emotions on the subjective quality of individual scenes in future thinking, as negative emotions potentially hinder the process of generating expectations (Hepburn et al., 2009). Meanwhile, personality disintegration, a state in which individuals perceive themselves as insignificant, can lead to feelings of despair and indifference toward the past, present, and future, and thus disrupt the complex metacognitive judgments necessary for making decisions about the future (Kinley et al., 2021). Since there is a close relationship between achieving accomplishments and realizing future goals (Lee et al., 2010), lacking personal accomplishment in nurses with burnout might lead to less specific future imagination.

Additionally, we found that nurses with burnout envisioned significantly fewer positive future events than nurses without burnout. This is further validated by the findings that nurses with burnout envisioned fewer happy events and events associated with relationships and achievement/mastery in their future, as well as the significant correlations between the scores of burnout and proportions of positive events, happy events and events on relationships and achievement/mastery. This observation could be attributed to the challenges that nurses with burnout encounter (e.g., serious emotional exhaustion and depersonalization, and decreased personal accomplishment), which making it difficult for them to envision positive scenarios during the chronic stress (Pines and Kanner, 1982). Given the evident significance of interpersonal relationships in nursing practice (D'Antonio et al., 2014), nurses are often required to manage complex interpersonal conflicts, which may contribute to job burnout (Duddle and Boughton, 2007; Carod-Artal and Vázquez-Cabrera, 2013). These findings suggest that nursing managers should focus on promoting positive emotional experiences and interpersonal relationships among nurses to prevent and alleviate job burnout (Lee and Jang, 2019).

However, no group difference was found concerning the negative future events between the nurses with burnout and without burnout. Previous studies showed that depressed people have decreased positive future expectations and increased negative expectations (Beck et al., 2006; Szőllősi et al., 2015). This is in line with our results of the deficits of positive envisioning, but does not agree with the absence of the enhancement of negative expectation. This inconsistent manifestations might be explained by the different features between depression and burnout (Brenninkmeyer et al., 2001). The core problem of burnout is the exhaustion of positive emotions, but depression is also accompanied by an increase in negative thinking. These findings suggested that interventions for burnout may require a different strategy from that for depression. Specifically, interventions for burnout should prioritize fostering positive thinking.

Few previous studies have addressed the problem of omission. We found that future thinking omission was severer among nurses with burnout than nurses without burnout. This result was further consolidated by the results concerning the levels of burnout, i.e., nurses with severe burnout were linked with more omissions than nurses with mild and moderate burnout. This suggested that nurses with burnout had difficulty when envisioning future events. Nurses with burnout generally experience chronic stress, which manifests as a low spirit and perceived dim prospects (Taormina and Law, 2000). They are often busy and struggle to balance work and family life. Over time, their image of the future becomes increasingly blurred, and their ability to imagine the future gradually diminishes. Therefore, in nursing practice, managers should prioritize training nurses in future thinking to alleviate burnout.

Furthermore, we found that the impairment of nurses’ future thinking became more serious as the levels of burnout increased. Nurses experiencing severe burnout face a dual challenge compared to nurses with mild or moderate burnout, they not only display greater ambiguity and omissions in their future thinking but also tend to perceive future events as less positive. These results suggested that when alleviating nurse burnout, it is essential to treat nurses suffering from different levels of burnout with various ways (Maslach and Leiter, 2016). Specifically, there is a critical need to focus on nurses experiencing severe burnout and provide targeted training to enhance their future thinking abilities (Szpunar and Schacter, 2013).

## Implications

In our study, we observed that nurses experiencing burnout demonstrated a significant impairment in constructing future scenarios and had difficulty envisioning future events, especially positive future events. These findings have vital implications for nursing managers. Studies have demonstrated that repeated mental simulations of future events can enhance an individual’s ability to vividly imagine the future with more details (Szpunar and Schacter, 2013). Nursing managers can utilize future thinking intervention to alleviate nurses’ burnout, such as future-oriented interventions (e.g., solution-focused brief therapy; Luo H. et al., 2019), future-oriented therapy (FOT) (Landkroon et al., 2022), and future-oriented occupational health services and workplace health promotion programs (Nucera et al., 2023). This intervention may help nurses with burnout develop a positive and hopeful future (Salmela-aro et al., 2004), which, in turn, can alleviate burnout, improve work efficiency, and enhance overall work quality. Furthermore, nursing managers can use positive psychological interventions, such as gratitude journal writing (Camero and Carrico, 2022), cultivating resilience (Alonazi et al., 2023), and focusing on personal strengths and values, to create a positive work environment that fosters positive emotions in nurses (Giorgi et al., 2016; Luo Y. H. et al., 2019).

## Limitations

Several limitations should be acknowledged in this study. First, the nurses in the present study were all female, whether the findings could be extended in male nurses remains to be investigated in future studies. Second, the nurses in our study came from a single hospital in eastern China, which might have its unique organizational culture and stressors that could potentially affect the nurses’ burnout and future thinking. Therefore, future research on the future thinking of nurses with job burnout can be conducted in a multi-center setting with a larger sample from different hospitals and even diverse countries with multi-culture. This will contribute to the generalizability of the findings. Third, we did not set a time limit for completing the SCEFT task, which might have influenced participants’ responses (Anderson and Dewhurst, 2009; Crane et al., 2013). Future research could consider implementing time constraints on future thinking to explore the effect.

## Conclusion

The current study found that nurses with burnout had impaired ability of future thinking. They could envision less specific and positive future events. In addition, as their symptoms of burnout progresses, the deficit in future thinking becomes worsen. The results of the present study indicated that burnout among nurses not only disturbs their present life but also have negative influence on their future thinking. Thus, our findings advocate effective interventions of positive future thinking to mitigate nurses’ burnout.

## Data availability statement

The raw data supporting the conclusions of this article will be made available by the authors, without undue reservation.

## Ethics statement

The studies involving humans were approved by the Ethics Committee of the School of Nursing Hangzhou Normal University (Approval no. 2022002). The studies were conducted in accordance with the local legislation and institutional requirements. The participants provided their written informed consent to participate in this study. Written informed consent was obtained from the individual(s) for the publication of any potentially identifiable images or data included in this article.

## Author contributions

BX, ZH, and HL contributed to conception and design of the study. BX, YF, YiZ, XL, and XY organized the database. BX, JZ, YZ, and SW performed the statistical analysis. BX wrote the first draft of the manuscript. YF and JZ wrote sections of the manuscript. All authors contributed to manuscript revision, read, and approved the submitted version.
