# Supplementary material for: Phenomenological characteristics of autobiographical future thinking in nurses with burnout: a case-control study
Source: Front Psychol. 2023 Oct 10;14:1216036. doi: 10.3389/fpsyg.2023.1216036 (PMC10597650; doi:10.3389/fpsyg.2023.1216036)
Supplement: Supplementary file 1 [file Data_Sheet_1.doc]

# Supplementary Information

**Table S1. Skewness and kurtosis values for each variable in SCEFT**

| **Item** | **Skewness** | **Kurtosis** |
| --- | --- | --- |
| Specific events | 1.02 | -0.17 |
| Extended events | 1.09 | 2.00 |
| Categorical events | 1.09 | 0.17 |
| Semantic associates | 0.27 | -0.99 |
| Omission | 1.21 | 0.76 |
| Positive | -0.16 | -1.26 |
| Negative | 2.41 | 5.54 |
| Neutral | 1.27 | 1.82 |
| Life-threatening events | 0 | 0 |
| Exploration/recreation | 0.96 | -0.20 |
| Relationships | 1.37 | 0.73 |
| Achievement/mastery | 0.96 | 0.66 |
| Guilt/shame | 7.95 | 67.68 |
| Drug/alcohol events | 0 | 0 |
| Hospitalization/stigmatization | 0 | 0 |
| Failure | 4.56 | 19.10 |
| Happy events | 0.64 | -0.26 |
| Career events | 0.47 | -0.70 |
| Neutral events | 1.09 | 1.11 |
| Not classifiable | 1.17 | 0.37 |

Note: SCEFT = Sentence Completion for Events in the Future Test.

Table S2. Demographic characteristics of the burnout and non-burnout groups (Mean(SD)).

| Demographic Characteristics |  | | Nurses with burnout  （n=70） | Nurses without burnout  （n=70） | *X^2^* | *p* |
| --- | --- | --- | --- | --- | --- | --- |
| education level | | associate degree bachelor degree  master degree | 3(4.3) | 5(7.1) | 0.53 | 0.767 |
|  |  |  | 66(94.3) | 64(91.4) |  |  |
|  |  |  | 1(1.4) | 1(1.4) |  |  |
| working seniority | | 1～6 years  7～15 years  16～25 years | 30(42.9) | 31(44.3) | 2.97 | 0.226 |
|  |  |  | 26(37.1) | 32(45.7) |  |  |
|  |  |  | 14(20.0) | 7(10.0) |  |  |
| employment form | | permanently employed | 43(61.4) | 36(51.4) | 1.42 | 0.233 |
|  |  | temporarily employed | 27(38.6) | 34(48.6) |  |  |
| professional title | | registered nurses | 13(18.6) | 18(25.7) | 5.06 | 0.168 |
|  | | senior nurse | 38(54.3) | 25(35.7) |  |  |
|  | | supervisor nurses | 17(24.3) | 23(32.9) |  |  |
|  | | co-chief nurses or above | 2(2.9) | 4(5.7) |  |  |
| working time with patients | | ≤8h | 26(37.1) | 36(51.4) | 3.56 | 0.169 |
|  |  | 8h~12h | 43(61.4) | 32(45.7) |  |  |
|  |  | ≥12h | 1(1.4) | 2(2.9) |  |  |

Table S3. Demographic characteristics of the three groups with different burnout levels.

| Demographic Characteristics |  | Mild burnout  (n=43) | Moderate burnout  (n=42) | Severe burnout  (n=43) | *X^2^* | *p* |
| --- | --- | --- | --- | --- | --- | --- |
| education level | associate degree bachelor degree  master degree | 2(4.7) | 2(4.8) | 3(7) | 2.31 | 0.679 |
|  |  | 41(95.3) | 40(95.2) | 39(90.7) |  |  |
|  |  | 0 | 0 | 1(2.3) |  |  |
| working seniority | 1～6 years  7～15 years  16～25 years | 14(32.6) | 20(47.6) | 18(41.9) | 2.40 | 0.662 |
|  |  | 24(55.8) | 17(40.5) | 21(48.8) |  |  |
|  |  | 5(11.6) | 5(11.9) | 4(9.3) |  |  |
| employment form | permanently employed | 26(60.5) | 16(38.1) | 18(41.9) | 4.92 | 0.085 |
|  | temporarily employed | 17(39.5) | 26(61.9) | 25(58.1) |  |  |
| professional title | registered nurses | 6(14.0) | 9(21.4) | 5(11.6) | 7.04 | 0.316 |
|  | senior nurse | 22(51.2) | 24(57.1) | 22(51.2) |  |  |
|  | supervisor nurses | 15(34.9) | 9(21.4) | 14(32.6) |  |  |
|  | co-chief nurses or above | 0 | 0 | 2(4.7) |  |  |
| working time with patients | ≤8h | 19(44.2) | 20(47.6) | 11(25.6) | 5.82 | 0.213 |
|  | 8h~12h | 23(53.5) | 22(52.4) | 31(72.1) |  |  |
|  | ≥12h | 1(2.3) | 0(2.9) | 1(2.3) |  |  |

Table S4. Intergroup nurses Demographic Characteristics (for sensitivity analysis).

| Demographic Characteristics |  | Nurses with burnout  （N=62） | Nurses without burnout  （N=78） | *X^2^* | *p* |
| --- | --- | --- | --- | --- | --- |
| education background | associate degree  bachelor degree  master degree | 2(3.2) | 5(6.4) | 0.46 | 0.326 |
|  |  | 60(96.8) | 73(93.6) |  |  |
|  |  | 1(1.4) | 1(1.4) |  |  |
| working seniority | 1～6years  7～15years  16～25years | 24(38.7) | 32(41.0) | 1.97 | 0.373 |
|  |  | 27(43.5) | 26(33.3) |  |  |
|  |  | 11(17.7) | 20(25.6) |  |  |
| employment form | public institution | 28(45.2) | 36(46.2) | 1.00 | 0.522 |
|  | contract nurses | 34(54.8) | 42(53.8) |  |  |
| professional title | nurse | 8(12.9) | 18(23.1) | 4.24 | 0.232 |
|  | nurse practitioner | 33(53.2) | 29(37.2) |  |  |
|  | nurse in charge | 18(29.0) | 26(33.3) |  |  |
|  | associate chief nurse | 3(4.8) | 5(6.4) |  |  |
| worked with patients | ≤8h | 23(37.1) | 38(48.7) | 2.82 | 0.197 |
|  | 8h~12h | 43(62.9) | 32(50.0) |  |  |
|  | ≥12h | 0 | 1(1.3) |  |  |

Table S5. Intra-group burnout nurses Demographic Characteristics (for sensitivity analysis).

| Demographic Characteristics |  | Mild burnout  (N=43) | Moderate burnout  (N=45) | Severe burnout  (N=40) | *X^2^* | *p* |
| --- | --- | --- | --- | --- | --- | --- |
| education background | associate degree bachelor degree  master degree | 2(4.7) | 3(6.7) | 2(5.0) | 2.09 | 1.000 |
|  |  | 41(95.3) | 41(91.1) | 38(95.0) |  |  |
|  |  | 0 | 1(2.2) | 0 |  |  |
| working seniority | 1～6years  7～15years  16～25years | 14(32.6) | 24(53.3) | 14(35.0) | 5.49 | 0.239 |
|  |  | 25(58.1) | 16(35.6) | 21(52.5) |  |  |
|  |  | 4(9.3) | 5(11.1) | 4(12.5) |  |  |
| employment form | public institution | 23(53.5) | 22(48.9) | 15(37.5) | 2.23 | 0.333 |
|  | contract nurses | 20(46.5) | 23(51.1) | 25(62.5) |  |  |
| professional title | nurse | 5(11.6) | 11(24.4) | 4(10.0) | 6.86 | 0.281 |
|  | nurse practitioner | 26(60.5) | 22(48.9) | 21(52.5) |  |  |
|  | nurse in charge | 12(27.9) | 12(26.7) | 13(32.5) |  |  |
|  | associate chief nurse | 0 | 0 | 2(5.0) |  |  |
| worked with patients | ≤8h | 19(44.2) | 16(35.6) | 15(37.5) | 2.27 | 0.745 |
|  | 8h~12h | 23(53.5) | 29(64.4) | 24(60.0) |  |  |
|  | ≥12h | 1(2.3) | 0 | 1(2.5) |  |  |

**Sensitivity analysis**

To ensure the methodological rigor of this study and verify the results, we conducted a sensitivity analysis. Specifically, we aimed to investigate whether our main findings remained consistent under different criteria for defining occupational burnout. We adjusted the standards for occupational burnout based on the norm for nurses in the Hangzhou city of China (Ye, Luo, & Jiang, 2008). Specifically, emotional exhaustion scores ≥ 27, depersonalization scores ≥ 8, and personal accomplishment scores ≤ 24 were used as the new criteria to define the burnout state in the sub-dimensions. Under these new criteria of burnout, the numbers of nurses included in the burnout group and no-burnout group, as well as the mild, moderate and severe burnout group were slightly different from that in the previous analyses. See the demographic characteristics of different groups in Table S4 and S5.

In the sensitivity analysis, we observed that even under these new criteria, nurses with burnout also exhibited significant differences in the proportion of imagined future events in SCEFT task compared to nurses without burnout (See the details in Table S6). Furthermore, we also found significant differences among nurses with mild, moderate and severe burnout (See the details in Table S7). Finally, most of the significant correlations between the scores of the sub-dimensions of burnout and the indices of future thinking remained (See the details in Table S8). Although there are some differences between the new results in the sensitivity analysis and the results in the main body of the manuscript, the main and important results were replicated in the sensitivity analysis. This enhances the credibility of our research findings and suggested that our results are convincing.

Table S6: Intergroup differences in future thinking between nurses with and without burnout (for sensitivity analysis).

| Item | Burnout  (N=64) | Without burnout  (N=76) | *t/Z* | *p* |
| --- | --- | --- | --- | --- |
| **Specificity** |  |  |  |  |
| Specific events | 0.14±0.21 | 0.36±0.32 | 4.74 | <0.001 |
| Extended events | 0.16±0.15 | 0.20±0.23 | 1.36 | 0.177 |
| Categorical events | 0.00(0.00,0.36) | 0.00(0.00,0.82) | -1.47 | 0.141 |
| Semantic associates | 0.45±0.25 | 0.35±0.28 | -2.16 | 0.033^*^ |
| Omission | 0.17±0.22 | 0.05±0.11 | -4.21 | <0.001^**^ |
| **Emotional valence** |  |  |  |  |
| Positive | 0.56±0.29 | 0.77±0.20 | 5.01 | <0.001^**^ |
| Negative | 0.00(0.00,0.45) | 0.00(0.00,0.36) | -2.14 | 0.032^*^ |
| Neutral | 0.18(0.00,0.45) | 0.18(0.00,0.55) | -0.12 | 0.903 |
| **Content** |  |  |  |  |
| Life-threatening events | 0 | 0 | / | / |
| Exploration/recreation | 0.12±0.06 | 0.12±0.08 | -0.14 | 0.886 |
| Relationships | 0.16±0.10 | 0.19±0.13 | 1.06 | 0.290 |
| Achievement/mastery | 0.16 ± 0.10 | 0.20 ± 0.13 | 0.57 | 0.100 |
| Guilt/shame | 0.00(0.00,0.09) | 0.00(0.00,0.09) | -0.56 | 0.572 |
| Drug/alcohol events | 0 | 0 | / | / |
| Hospitalization/stigmatization | 0 | 0 | / | / |
| Failure | 0.00(0.00,0.09) | 0.00(0.00,0.09) | 0.00 | 1.000 |
| Happy events | 0.20 ± 0.11 | 0.27 ± 0.18 | 2.73 | 0.007^*^ |
| Career events | 0.20 ± 0.12 | 0.22 ± 0.12 | 1.22 | 0.224 |
| Neutral events | 0.09±0.11 | 0.08±0.10 | -0.48 | 0.631 |
| Not classifiable | 0.30±0.22 | 0.14±0.12 | -5.05 | <0.001^**^ |

Note:For data that does not conform to a normal distribution, the form of "median (min, max)" was used to describe them. For data that conforms to a normal distribution, the "mean ± SD" was adopted to describe them. **p*< 0.05. ***p* < 0.001.

Table S7: Intragroup differences in future thinking among nurses with different burnout levels (for sensitivity analysis).

| Item | Mild  (N=43) | Moderate  (N=45) | Severe  (N=40) | *H/F* | *p* |
| --- | --- | --- | --- | --- | --- |
| **Specificity** |  |  |  |  |  |
| Specific events | 0.20±0.28 | 0.14±0.13 | 0.08±0.09 | 4.158 | 0.018^*^ |
| Extended events | 0.18(0.00,0.55) | 0.18(0.00,0.45) | 0.18(0.00,0.36) | 14.13 | 0.001^**^ |
| Categorical events | 0.00(0.00,0.82) | 0.00(0.00,0.45) | 0.00(0.00,0.82) | 0.09 | 0.955 |
| Semantic associates | 0.36(0.00,0.91) | 0.36(0.00,0.73) | 0.36(0.00,1.00) | 0.01 | 0.994 |
| Omission | 0.09(0.00,0.55) | 0.09(0.00,0.82) | 0.09(0.00,0.82) | 28.74 | <0.001^**^ |
| **Emotion valence** |  |  |  |  |  |
| Positive | 0.55(0.00,1.00) | 0.55(0.00,1.00) | 0.55(0.00,1.00) | 12.44 | 0.002^*^ |
| Negative | 0.00(0.00,0.18) | 0.00(0.00,0.36) | 0.00(0.00,0.27) | 0.60 | 0.741 |
| Neutral | 0.18(0.00,0.45) | 0.18(0.00,0.91) | 0.18(0.00,0.45) | 4.07 | 0.131 |
| **Content** |  |  |  |  |  |
| Life-threatening events | 0 | 0 | / | / | / |
| Exploration/recreation | 0.00(0.00,0.27) | 0.00(0.00,0.27) | 0.00(0.00,0.18) | 8.87 | 0.012^*^ |
| Relationships | 0.14±0.12 | 0.15±0.11 | 0.10±0.09 | 2.748 | 0.068 |
| Achievement/mastery | 0.09(0.00,0.55) | 0.09(0.00,0.55) | 0.09(0.00,0.55) | 7.08 | 0.029 |
| Guilt/shame | 0 | 0 | / | / | / |
| Drug/alcohol events | 0 | 0 | / | / | / |
| Hospitalization/stigmatization | 0 | 0 | / | / | / |
| Failure | 0.09 | 0.09 | 0.09 | 0.000 | 1.000 |
| Happy events | 0.18(0.00,0.64) | 0.18(0.00,0.45) | 0.18(0.00,0.55) | 2.68 | 0.262 |
| Career events | 0.09(0.00,0.45) | 0.09(0.00,0.73) | 0.09(0.00,0.45) | 0.05 | 0.978 |
| Neutral events | 0.00(0.00,0.27) | 0.00(0.00,0.55) | 0.00(0.00,0.45) | 0.39 | 0.824 |
| Not classifiable | 0.18(0.00,0.82) | 0.18(0.00,0.82) | 0.18(0.00,0.82) | 6.45 | 0.040^*^ |

Note: For data that does not conform to a normal distribution, the form of "median (min, max)" was used to describe them. For data that conforms to a normal distribution, the "mean ± SD" was adopted to describe them. **p*< 0.05. ***p* < 0.001.

Table S8: Correlations of nurses’ burnout and future thinking (for sensitivity analysis).

| Item | 1 | 2 | 3 | 4 | 5 | 6 | 7 |
| --- | --- | --- | --- | --- | --- | --- | --- |
| 1 | 1 |  |  |  |  |  |  |
| 2 | 0.500^**^ | 1 |  |  |  |  |  |
| 3 | -0.661^**^ | -0.433^**^ | 1 |  |  |  |  |
| 4 | -0.279^**^ | -0.257^**^ | 0.372^**^ | 1 |  |  |  |
| 5 | 0.293^**^ | 0.119 | -0.208^**^ | -0.256^**^ | 1 |  |  |
| 6 | -0.379^*^ | -0.168^*^ | 0.299^**^ | 0.322^**^ | -0.580^**^ | 1 |  |
| 7 | -0.237^**^ | -0.143 | 0.137 | 0.138 | -0.409^**^ | 0.456^**^ | 1 |

Notes: 1=Emotional exhaustion, 2=Depersonalization, 3=Personal accomplishment, 4=Specific events, 5=Omissions, 6=Positive events, 7=Events on relationships, 8=Events on achievement/mastery, 9=Happy events. Note: **p*< 0.05. ***p* < 0.001.
